# Associations of Small Fiber Neuropathy with Geriatric Nutritional Risk Index and Arterial Stiffness in Hemodialysis

**DOI:** 10.1155/2020/1694218

**Published:** 2020-05-19

**Authors:** Mei-Chuan Kuo, Jiun-Chi Huang, Pei-Yu Wu, Hsiu-Chin Mai, Szu-Chia Chen, Yi-Wen Chiu, Jer-Ming Chang, Hung-Chun Chen

**Affiliations:** ^1^Division of Nephrology, Department of Internal Medicine, Kaohsiung Medical University Hospital, Kaohsiung Medical University, Kaohsiung, Taiwan; ^2^Faculty of Renal Care, College of Medicine, Kaohsiung Medical University, Kaohsiung, Taiwan; ^3^Department of Internal Medicine, Kaohsiung Municipal Siaogang Hospital, Kaohsiung Medical University, Kaohsiung, Taiwan; ^4^Faculty of Medicine, College of Medicine, Kaohsiung Medical University, Kaohsiung, Taiwan; ^5^Graduate Institute of Clinical Medicine, College of Medicine, Kaohsiung Medical University, Kaohsiung, Taiwan; ^6^Department of Nursing, Kaohsiung Municipal Siaogang Hospital, Kaohsiung Medical University, Kaohsiung, Taiwan

## Abstract

**Background:**

Peripheral neuropathy is a common neurological complication in uremic patients, and quantitative sensory testing (QST) is effective for diagnosis of small fiber neuropathy. Malnutrition and arterial stiffness are prevalent in patients undergoing hemodialysis (HD). The associations of small fiber neuropathy with nutritional status and arterial stiffness remain uncertain in maintenance HD patients.

**Methods:**

A total of 152 HD patients were included. Geriatric nutritional risk index (GNRI), an indicator of nutritional status, was calculated by serum albumin and actual and ideal body weight. Arterial stiffness was defined as brachial-ankle pulse wave velocity (baPWV) > 1400 cm/s. Small fiber neuropathy was assessed by an abnormal QST threshold of cold and warm sensation in patients' hands or feet. Multivariate forward logistic regression analysis was performed to examine the associations among abnormal QST threshold, GNRI, and arterial stiffness.

**Results:**

baPWV and prevalence of abnormal QST threshold were significantly higher in diabetic patients. Multivariate logistic analyses revealed that older age (OR, 1.081; 95% CI, 1.026–1.139, *p* = 0.003) and male gender (OR, 4.450; 95% CI, 1.250–15.836, *p* = 0.021) were associated with abnormal warm threshold of hands. Furthermore, diabetes (OR, 3.966; 95% CI, 1.351–11.819, *p* = 0.012) and lower GNRI (per 1 unit increase, OR, 0.935, 95% CI, 0.887–0.985, *p* = 0.012) were associated with abnormal cold threshold of feet. Arterial stiffness (OR, 5.479, 95% CI, 1.132–22.870, *p* = 0.020) and higher calcium-phosphorus product (OR, 1.071, 95% CI, 1.013–1.132, *p* = 0.015) were associated with abnormal warm threshold of feet.

**Conclusions:**

Lower GNRI and arterial stiffness were significantly associated with small fiber neuropathy in patients undergoing HD. Malnutrition risk and vascular factors might play important roles in small fiber neuropathy among patients undergoing HD.

## 1. Introduction

Patients with end-stage renal disease (ESRD) often suffer from neurological complications, thereby contributing to morbidity and mortality [1–3]. Peripheral neuropathy is the most commonly reported neurological complication associated with chronic renal failure [4], with an incidence rate of more than 60% in patients on dialysis. It can affect sensory, motor, and cranial nerves and is characterized by axonal degeneration and demyelination [5]. Furthermore, uremic neuropathy is typically a distal, symmetric, and predominantly axonal type which affects the legs more than the arms [1, 2]. Lindblom and Tegner reported abnormalities in thermal sensation in 30% of patients with ESRD and concluded that small fiber neuropathy may be a distinct entity [6]. Small nerve fibers were traditionally thought to be invisible as they could not be detected in routine nerve conduction studies, leading to underestimation of small fiber neuropathy and physicians' overlook. Skin biopsy for detecting altered interepidermal nerve fiber density remains the gold standard in diagnosing small fiber neuropathy, but it is still an invasive approach [7, 8]. Quantitative sensory testing (QST) is a noninvasive, psychophysical examination of small fiber functions through assessment of thresholds to thermal and cold signals [9]. Furthermore, QST has been shown to be useful in diagnosis of small fiber neuropathy [10–12], with a reported diagnostic sensitivity ranging from 60% to 85% [13–16].

Small fiber neuropathy is characterized by the presence of abnormal thermal thresholds in the distal limbs, linking to decreased quality of life in affected individuals, and is often neglected in clinical practice because of a paucity of readily available diagnostic methods [17]. Nutritional aspects and microvascular dysregulation were previously advocated as the potential causes of small fiber neuropathy [11, 18, 19]. In particular, arterial stiffness and malnutrition are common among patients undergoing hemodialysis (HD) and play their vital roles in prognostic significance [20–24]. The geriatric nutritional risk index (GNRI) is a useful and accurate indicator for the assessment of nutritional status in maintenance HD patients [25, 26]. Several studies have suggested that GNRI and arterial stiffness are associated with cognitive impairment, frailty, and inflammation [27–31]. However, the relationships of malnutrition risk and arterial stiffness with small fiber neuropathy remain not clearly understood and have never been investigated. Therefore, the aim of this study is to examine the associations of malnutrition risk and arterial stiffness, assessed by GNRI and brachial-ankle pulse wave velocity (baPWV), with small fiber neuropathy in patients receiving HD.

## 2. Materials and Methods

### 2.1. Study Patients

In the present study, we enrolled all patients (*n* = 170) undergoing thrice weekly maintenance HD treatment for more than 3 months at a dialysis unit of a regional hospital in Taiwan in April 2014. Patients who refused to undergo QST or baPWV examinations (*n* = 15) and had bilateral below knee amputations (*n* = 3) were excluded from the study. This study was approved by the Institutional Review Board of Kaohsiung Medical University Hospital. All study participants provided their written informed consent. The methods were carried out in accordance with the approved guidelines.

### 2.2. Measurement of QST for Assessment of Small Fiber Neuropathy

A Medoc TSA-II Neurosensory Analyzer was used for the QST [32]. The patients were seated comfortably in a quiet room with an ambient temperature of 24–25°C. The testing areas of the QST were the dorsolateral border of the feet for the lower limbs and the hypothenar eminence of the thumbs for the upper limbs. Cold and warm sensations were then tested after the test had been carefully explained to the patients, including the need to react promptly to any change in temperature. The test was performed by placing a 30 mm × 30 mm thermode on the testing areas of the skin. Four threshold temperature values for each testing area were recorded and averaged for analysis. If the sensation was identified incorrectly, this was also recorded. Based on values obtained from a control population, the mean threshold temperature ± 2 standard deviation (SD) for each testing area was considered as the upper (or lower) limit of normal. Small fiber neuropathy was defined as the abnormalities of cold or warm threshold in one of the testing areas (either hands or feet) using QST [9, 11].

### 2.3. Measurement of baPWV for Assessment of Arterial Stiffness

The baPWV was measured 10–30 minutes before HD session using an automatic waveform analyzer (VP-1000, Colin, Komaki, Japan) for each patient. The baPWV value was calculated as the transmission distance divided by the transmission time. The highest of bilateral baPWV values was used as the representative value for analysis. Arterial stiffness was defined as baPWV > 1400 cm/s [33].

### 2.4. Calculation of the GNRI

The GNRI was calculated according to baseline serum albumin level and body weight as follows: GNRI = [14.89 × albumin (g/dL)] + [41.7 × (body weight/ideal body weight)] [34]. The ideal body weight in the present study was defined as the value calculated from the height and a body mass index (BMI) of 22 [35]. If the patient's body weight was greater than the ideal body weight, body weight/ideal body weight was set to 1 [27].

### 2.5. Demographic, Medical, and Laboratory Data

Each patient's overnight fasting blood samples were obtained within 1 month of study enrollment for laboratory tests using an autoanalyzer (Roche Diagnostics GmbH, D-68298 Mannheim COBAS Integra 400). Kt/V was evaluated based on the Daugirdas formula to assess the efficiency of HD treatment [36]. Demographic and medical information, including age, gender, smoking history (ever *vs*. never), and comorbid conditions, were obtained from study patients' medical records and interviews.

### 2.6. The Primary and Secondary Outcomes

The primary outcome of this study was to elucidate the effects of diabetes and arterial stiffness on thermal threshold in QST among HD patients. The secondary outcome was to identify the determinants of abnormal QST threshold as well as the roles of nutritional risk and arterial stiffness in small fiber neuropathy.

### 2.7. Statistical Analysis

Statistical analysis was performed using SPSS for Windows version 19.0 (SPSS Inc., Chicago, IL, USA). Data are expressed as percentage, mean ± SD, or median (25^th^–75^th^ percentile) for the duration of dialysis and serum triglycerides. Between-group differences were analyzed using the chi-square test for categorical variables and the independent *t*-test for continuous variables. Multivariate forward logistic regression analysis was performed to identify the factors associated with abnormal QST threshold, with adjustment for age, gender, duration of dialysis, smoking history, diabetes mellitus, baPWV > 1400 cm/s, GNRI, hemoglobin, calcium-phosphorus (Ca × P) product, and Kt/V. A difference was considered significant for the *p* value < 0.05.

## 3. Results

A total of 152 patients (78 men and 74 women, mean age 60.7 ± 10.7 years) were included in the present study. The mean baPWV was 1925 ± 526 cm/s, and the mean GNRI was 103.4 ± 9.0.

### 3.1. Comparison of QST Threshold between Diabetic and Nondiabetic Patients

As diabetes mellitus (DM) is one of the major risk factors of neurologic complications, the comparison of baseline characteristics and thermal threshold on QST between diabetic and nondiabetic patients is summarized in Table 1. Diabetic patients were more likely to be older in age and had shorter duration of dialysis, higher baPWV, lower cold threshold, and higher warm threshold among the QST of both hands and feet. Diabetic patients significantly had higher prevalence of abnormal cold and warm threshold on QST, except for warm threshold of feet, when compared to nondiabetic patients.

### 3.2. Comparison of QST Threshold between Patients with baPWV ≤ 1400 cm/s and >1400 cm/s

**Figure 1:**
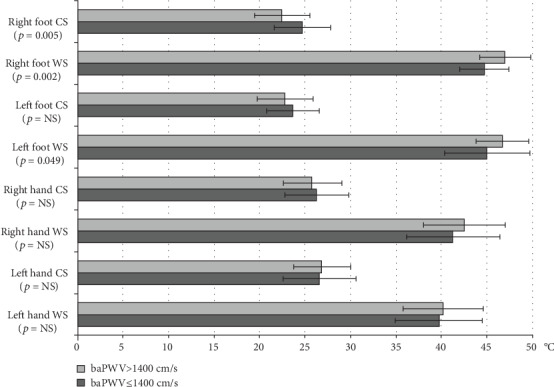
Comparison of QST threshold between patients with baPWV > 1400 cm/s and ≤1400 cm/s. CS: cold threshold; WS: warm threshold.

Among QST results, patients with baPWV > 1400 cm/s had significantly lower cold threshold of the right foot (*p* = 0.005) and significantly higher warm threshold of the right (*p* = 0.002) and left feet (*p* = 0.049) in comparison to QST in patients with baPWV ≤ 1400 cm/s (Figure 1).

### 3.3. Determinants of Abnormal QST Threshold

The study patients were then stratified into two groups according to normal or abnormal QST threshold. The comparison of baseline characteristics between patients with normal or abnormal QST threshold of the hands is shown in Table 2. The prevalence of abnormal cold and warm sensation in the hands was 73.0% and 88.8%, respectively. Compared to patients with normal cold sensation, those with abnormal cold sensation in the hands had older age, shorter duration of dialysis, higher prevalence of DM, and higher baPWV. Moreover, compared to patients with normal warm sensation, patients with abnormal warm sensation in the hands had higher prevalence of male gender.

The comparison of baseline characteristics between patients with normal or abnormal QST threshold of the feet was demonstrated in Table 3. The prevalence of abnormal cold and warm sensation in the feet was 84.9% and 89.5%, respectively. Compared to patients with normal cold sensation, those with abnormal cold sensation in the feet had older age, higher prevalence of DM, higher baPWV, and lower GNRI. Furthermore, compared to patients with normal warm sensation, patients with abnormal warm sensation in the feet had higher Ca × P product.

Table 4 shows the determinants of abnormal QST threshold in our study patients using multivariate forward logistic regression analysis. In the multivariate analysis (adjusted for age, gender, duration of dialysis, smoking history, DM, baPWV > 1400 cm/s, GNRI, hemoglobin, Ca × P product, and Kt/V), old age (per 1 year; odds ratio (OR), 1.083; 95% confidence interval (CI), 1.042 to 1.126; *p* < 0.001) was independently associated with abnormal cold threshold of the hands. Old age (per 1 year; OR, 1.081; 95% CI 1.026 to 1.139; *p* = 0.003) and male gender (OR, 4.450; 95% CI, 1.250 to 15.836; *p* = 0.021) were significantly associated with abnormal warm threshold of the hands. Furthermore, DM (OR, 3.996; 95% CI, 1.351 to 11.819; *p* = 0.012) and low GNRI (per 1 unit; OR, 0.935; 95% CI, 0.887 to 0.985; *p* = 0.012) were significantly associated with abnormal cold threshold of the feet. Finally, baPWV > 1400 cm/s (OR, 5.479; 95% CI, 1.312 to 22.870; *p* = 0.020) and high Ca × P product (per 1 mg^2^/dL^2^; OR, 1.071; 95% CI, 1.013 to 1.132; *p* = 0.015) were independently associated with abnormal warm threshold of the feet.

## 4. Discussion

In this study, we found that the prevalence of abnormal QST threshold and baPWV were significantly higher in diabetic HD patients. Furthermore, we examined factors associated with small fiber neuropathy. In multivariate analysis, older age was associated with abnormal cold and warm threshold of the hands; DM and lower GNRI were significantly associated with abnormal cold threshold of the feet, whereas baPWV > 1400 cm/s and higher Ca × P product were significantly associated with abnormal warm threshold of the feet in HD patients.

The first important finding of this study is that patients undergoing HD with baPWV > 1400 cm/s had significant higher warm threshold and lower cold threshold in the feet on QST. In addition, patients with abnormal thermal threshold in the hands and abnormal cold threshold in the feet had significantly higher baPWV level than those with normal QST threshold. This suggests that arterial stiffness might be an important factor contributed to abnormalities of small fiber function in HD patients. Our findings are in line with previous studies showing an independent association between pulse wave velocity and peripheral neuropathy in patients with DM [37–39]. Elevated baPWV evinces structural changes of the arterial wall, medial smooth muscle calcification, and breaks in elastic fibers in ESRD [40]. Increased arterial stiffness might lead to damage of the microcirculation, such as vasa nervorum, through increasing the transmission of detrimental pulsatile pressure waves [41]. Furthermore, emerging evidence suggests independent association of arterial stiffness with galectin-3 among HD patients [42]. Galectin-3 has been recently considered as a key molecule in the neural functions and nerve regeneration, and inhibition of galectin-3 could attenuate neuropathic pain after peripheral nerve injury [43, 44]. Our results may bring additional insight into the role of arterial stiffness in small fiber neuropathy in maintenance HD patients.

Another important finding of this study is that a low GNRI was associated with an abnormal QST threshold. The pathogenesis of small fiber neuropathy in patients with ESRD is complex and remains not fully understood. Malnutrition is associated with increased level of tumor necrosis factor-*α* (TNF-*α*) in patients undergoing dialysis [45]. TNF-*α* has been shown to act a central role in the development of inflammatory demyelination [46]. Moreover, uremic toxins, combined with oxidative stress-related free radical activity and hyperkalemia, have been reported to cause motor, sensory, and autonomic nerve damage, making these as potentially causative factors in the development of uremic neuropathy [4]. Therefore, malnutrition risk might be involved in the pathogenesis of small fiber neuropathy in this patient population.

Abnormalities of homeostasis among calcium, phosphate, and parathyroid hormone are quite common in patients with ESRD, and increased Ca × P product promotes vascular calcification [47]. Recent studies demonstrated that impaired intracellular calcium balance is implicated in the development of diabetic polyneuropathy [48], while calcimimetic can deter the progression of neuropathy by ameliorating inflammation, apoptosis, and autophagy through increased expression of the calcium-sensing receptors [49]. In this study, we found that a higher level of Ca × P product was associated with an abnormal QST threshold, suggesting that vascular calcification and homeostasis of calcium and phosphate may have their roles mediating functional abnormalities in small nerve fibers in patients undergoing HD.

Maintenance HD patients often have certain neurological complications, including small fiber neuropathy, which can be linked to frailty and decreased quality of life. To the best of our knowledge, this is the first study to investigate the associations of GNRI and baPWV with small fiber neuropathy in HD patients. Nonetheless, there are several limitations in the present study. First, the number of study patients is relatively small. Second, this study was cross-sectional in design; therefore, the causal relationship and long-term clinical outcomes could not be confirmed. Further prospective studies and more participants are warranted to examine the impacts of nutrition and vascular factors as well as their involving roles in small fiber neuropathy. Third, confirmation of small fiber neuropathy using skin biopsy was lacking. Although skin biopsy is able to detect altered interepidermal nerve fiber density, it remains an invasive approach.

## 5. Conclusion

Our results demonstrated that small fiber neuropathy was associated with a lower GNRI and baPWV > 1400 cm/s. Furthermore, older age, DM, and a higher level of Ca × P product were associated with an abnormal QST threshold. Physicians should devote more attention toward maintenance HD patients with malnutrition risk and arterial stiffness to early diagnose small fiber neuropathy, prevent serious neurological complications, and improve quality of life.

## Figures and Tables

**Table 1:** Comparison of cold and warm threshold and baPWV between diabetic and nondiabetic patients.

Variables	Diabetic (*n* = 72)	Nondiabetic (*n* = 80)	*p*
Age (year)	62.9 ± 9.1	58.8 ± 11.7	0.018
Male gender (%)	55.6	47.5	0.321
Duration of dialysis (year)	3.5 (1.2–7.6)	9.1 (4.9–13.1)	<0.001
baPWV (cm/s)	2091 ± 545	1774 ± 461	<0.001
Body mass index (kg/m^2^)	24.7 ± 3.2	23.6 ± 4.3	0.074
GNRI	104.2 ± 7.4	102.7 ± 10.2	0.289
Cold threshold of the left hand (°C)	26.4 ± 3.2	27.4 ± 3.2	0.043
Warm threshold of the left hand (°C)	41.6 ± 4.9	38.7 ± 3.5	<0.001
Cold threshold of the right hand (°C)	25.0 ± 3.2	26.7 ± 3.0	0.001
Warm threshold of the right hand (°C)	43.9 ± 4.5	40.8 ± 4.2	<0.001
Cold threshold of the left foot (°C)	21.8 ± 2.7	23.9 ± 3.0	<0.001
Warm threshold of the left foot (°C)	47.8 ± 3.2	45.3 ± 2.7	<0.001
Cold threshold of the right foot (°C)	21.8 ± 2.7	23.7 ± 3.2	<0.001
Warm threshold of the right foot (°C)	47.8 ± 2.6	45.8 ± 2.8	<0.001
Abnormal cold threshold of the hands (%)	81.9	65.0	0.019
Abnormal warm threshold of the hands (%)	95.8	82.5	0.009
Abnormal cold threshold of the feet (%)	93.1	77.5	0.008
Abnormal warm threshold of the feet (%)	94.4	85.0	0.058

Abbreviations: baPWV: brachial-ankle pulse wave velocity; GNRI: geriatric nutritional risk index.

**Table 2:** Comparison of baseline characteristics between patients with normal or abnormal QST threshold of the hands.

Parameters	Cold threshold			Warm threshold		
	Normal (*n* = 41)	Abnormal (*n* = 111)	*p*	Normal (*n* = 17)	Abnormal (*n* = 135)	*p*
Age (year)	54.8 ± 10.8	62.9 ± 9.8	<0.001	53.7 ± 8.3	61.6 ± 10.7	0.004
Male gender (%)	48.8	52.3	0.704	23.5	54.8	0.015
Duration of dialysis (year)	8.8 (4.7–13.3)	6.1 (2.0–10.3)	0.023	10.7 (5.1–13.3)	6.3 (2.3–10.4)	0.066
Smoking history (%)	34.1	39.6	0.536	17.6	40.7	0.109
Diabetes mellitus (%)	31.7	53.2	0.019	17.6	51.1	0.009
Coronary artery disease (%)	2.4	11.7	0.114	0	10.4	0.369
baPWV (cm/s)	1766 ± 406	1982 ± 553	0.027	1672 ± 367	1956 ± 535	0.041
Body mass index (kg/m^2^)	24.4 ± 4.6	23.9 ± 3.5	0.495	23.6 ± 3.6	24.1 ± 3.9	0.603
GNRI	104.9 ± 11.0	102.8 ± 8.1	0.217	101.9 ± 9.9	103.6 ± 8.9	0.480
Laboratory parameters						
Albumin (g/dL)	3.9 ± 0.4	3.9 ± 0.3	0.155	3.8 ± 0.4	3.9 ± 0.3	0.552
Triglycerides (mg/dL)	125 (94–273)	139 (91–217)	0.340	149 (90–249)	131 (91–219)	0.707
Total cholesterol (mg/dL)	185.3 ± 34.6	179.7 ± 41.1	0.439	190.9 ± 40.0	180.0 ± 39.4	0.284
Hemoglobin (g/dL)	10.7 ± 1.0	10.6 ± 1.3	0.828	10.5 ± 1.3	10.7 ± 1.2	0.685
Total calcium (mg/dL)	9.5 ± 1.1	9.3 ± 1.0	0.362	9.0 ± 1.2	9.4 ± 1.0	0.241
Phosphorus (mg/dL)	4.6 ± 1.2	4.5 ± 1.0	0.846	4.7 ± 1.6	4.5 ± 1.0	0.490
Ca × P product (mg^2^/dL^2^)	43.2 ± 11.7	42.2 ± 11.1	0.624	43.3 ± 15.4	42.4 ± 10.7	0.755
Kt/V (Daugirdas)	1.6 ± 0.3	1.6 ± 0.3	0.695	1.7 ± 0.3	1.6 ± 0.3	0.070

Abbreviations: baPWV: brachial-ankle pulse wave velocity; GNRI: geriatric nutritional risk index; Ca × P product: calcium-phosphorus product.

**Table 3:** Comparison of baseline characteristics between patients with normal or abnormal QST threshold of the feet.

Parameters	Cold threshold			Warm threshold		
	Normal (*n* = 23)	Abnormal (*n* = 129)	*p*	Normal (*n* = 16)	Abnormal (*n* = 136)	*p*
Age (year)	55.6 ± 10.5	61.7 ± 10.5	0.011	56.6 ± 12.7	61.2 ± 10.4	0.100
Male gender (%)	65.2	48.8	0.148	23.5	54.8	0.242
Duration of dialysis (year)	9.0 (3.4–11.8)	6.5 (2.3–10.8)	0.614	9.9 (2.9–12.6)	6.4 (2.4–10.6)	0.218
Smoking history (%)	47.8	36.4	0.300	31.3	39.0	0.548
Diabetes mellitus (%)	21.7	51.9	0.008	25.0	50.0	0.068
Coronary artery disease (%)	0	10.9	0.130	0	10.3	0.364
baPWV (cm/s)	1691 ± 352	1966 ± 541	0.023	1876 ± 711	1931 ± 502	0.694
Body mass index (kg/m^2^)	25.9 ± 6.1	23.7 ± 3.2	0.011	24.3 ± 3.5	24.0 ± 3.9	0.797
GNRI	107.9 ± 12.6	102.6 ± 7.9	0.008	103.7 ± 9.3	103.3 ± 9.0	0.873
Laboratory parameters						
Albumin (g/dL)	3.9 ± 0.3	3.9 ± 0.3	0.242	3.9 ± 0.3	3.9 ± 0.3	0.919
Triglycerides (mg/dL)	125 (87–304)	139 (92–219)	0.309	108.5 (88–229)	139 (91.3–219)	0.928
Total cholesterol (mg/dL)	182.4 ± 27.3	181.1 ± 41.3	0.885	171.7 ± 34.8	182.4 ± 39.9	0.307
Hemoglobin (g/dL)	10.9 ± 1.2	10.6 ± 1.2	0.208	10.9 ± 1.5	10.6 ± 1.2	0.435
Total calcium (mg/dL)	9.3 ± 1.1	9.3 ± 1.0	0.844	9.1 ± 1.1	9.3 ± 1.0	0.371
Phosphorus (mg/dL)	4.7 ± 1.4	4.5 ± 1.0	0.625	4.1 ± 1.0	4.6 ± 1.1	0.058
Ca × P product (mg^2^/dL^2^)	43.1 ± 13.0	42.3 ± 11.0	0.753	37.0 ± 9.6	43.1 ± 11.3	0.041
Kt/V (Daugirdas)	1.6 ± 0.3	1.6 ± 0.3	0.557	1.6 ± 0.3	1.6 ± 0.3	0.857

Abbreviations: baPWV: brachial-ankle pulse wave velocity; GNRI: geriatric nutritional risk index; Ca × P product: calcium-phosphorus product.

**Table 4:** Determinants of abnormal QST threshold using multivariate forward logistic regression analysis.

Parameters	Multivariate (forward)	
	OR (95% CI)	*p*
Abnormal cold threshold of the hands		
Age (per 1 year)	1.083 (1.042–1.126)	<0.001
Abnormal warm threshold of the hands		
Age (per 1 year)	1.081 (1.026–1.139)	0.003
Male gender (*vs.* female)	4.450 (1.250–15.836)	0.021
Abnormal cold threshold of the feet		
Diabetes mellitus	3.996 (1.351–11.819)	0.012
GNRI (per 1 unit)	0.935 (0.887–0.985)	0.012
Abnormal warm threshold of the feet		
baPWV > 1400 cm/s	5.479 (1.312–22.870)	0.020
Ca × P product (per 1 mg^2^/dL^2^)	1.071 (1.013–1.132)	0.015

Values expressed as odds ratio (OR) and 95% confidence interval (CI). Adjusted for age, gender, duration of dialysis, smoking history, diabetes mellitus, baPWV > 1400 cm/s, GNRI, hemoglobin, Ca × P product, and Kt/V.

## Data Availability

The data supporting the findings of the present study are available within the article or are available from the corresponding author upon reasonable request.
